# Purification and characterization of FtsZ from the citrus canker pathogen *Xanthomonas citri* subsp. *citri*

**DOI:** 10.1002/mbo3.706

**Published:** 2018-08-07

**Authors:** Malgorzata M. Kopacz, André S. G. Lorenzoni, Carlos R. Polaquini, Luis O. Regasini, Dirk‐Jan Scheffers

**Affiliations:** ^1^ Department of Molecular Microbiology Groningen Biomolecular Sciences and Biotechnology Institute University of Groningen Groningen The Netherlands; ^2^ Laboratory of Antibiotics and Chemotherapeutics Department of Chemistry and Environmental Sciences Institute of Biosciences, Humanities and Exact Sciences São Paulo State University (UNESP) São José do Rio Preto SP Brazil; ^3^ Present address: Department of Chemical Engineering Biotechnology and Environmental Technology University of Southern Denmark Odense M Denmark

**Keywords:** antibacterials, cell division, FtsZ, gallates, gallic acid, GTPase, membrane permeabilization

## Abstract

*Xanthomonas citri* subsp. *citri* (Xac) is the causative agent of citrus canker, a plant disease that significantly impacts citriculture. In earlier work, we showed that alkylated derivatives of gallic acid have antibacterial action against Xac and target both the cell division protein FtsZ and membrane integrity in *Bacillus subtilis*. Here, we have purified native XacFtsZ and characterized its GTP hydrolysis and polymerization properties. In a surprising manner, inhibition of XacFtsZ activity by alkyl gallates is not as strong as observed earlier with *B. subtilis* FtsZ. As the alkyl gallates efficiently permeabilize Xac membranes, we propose that this is the primary mode of antibacterial action of these compounds.

## 1 INTRODUCTION

In the search for new antibacterial agents and targets for antimicrobials, many groups have focused on bacterial cell division as a process that is unique to prokaryotic cells and which involves proteins that often have no direct counterparts in eukaryotes. The cell division protein that has been most widely investigated in this respect is the extremely well‐conserved protein FtsZ, a (distant) homolog of tubulin that is absolutely critical for cell division (Den Blaauwen, Andreu, & Monasterio, 2014; Li & Ma, 2015; Panda et al., 2016). We have recently characterized the mode of action of alkyl gallates, antimicrobial agents that target both FtsZ and membrane integrity in the Gram‐positive model organism *Bacillus subtilis* (Król et al., 2015). Our ultimate aim, however, was to investigate the activity of alkyl gallates against FtsZ from the Gram‐negative plant pathogen *Xanthomonas citri* subsp. *citri* (Xac), as this is the organism that was used to identify the antibacterial activity of the alkylated derivatives of gallic acid and the pathogen we would like to combat (Silva et al., 2013).

Xac is the causal agent of Asiatic citrus canker, a severe plant disease that affects citrus crops, decreases fruit production, and causes economic losses (Gottwald, Graham, & Schubert, 2002). Asiatic citrus canker affects all the commercially important citrus species and cultivars in use (Gottwald et al., 2002). Infected trees exhibit brownish crater‐like lesions on aerial tissues that are sometimes surrounded by chlorotic halos. The disease leads to defoliation and premature fruit drop, which with time, decreases citrus fruit production. The disease is currently present in South and North America, Asia, Africa, and Oceania (Behlau, Fonseca, & Belasque, 2016; Davis et al., 2015; Leduc et al., 2015; Stover et al., 2014). The state of São Paulo, Brazil, the largest producer of concentrated orange juice in the world, has now been declared an area of Risk Mitigation System according to current regulation (MAPA, Brazil, Normativa 37, September 2016), meaning the disease is now considered endemic in this area. Infection control is subject to pressure from the orange production chain and currently involves plantation of less susceptible citrus cultivars, the use of windbreaks to avoid bacteria spreading by wind and rain as well as to prevent wind‐induced damage to trees which also facilitates infection, and the eradication of symptomatic trees along with spraying copper‐containing bactericides in a radius of 30 m of the symptomatic tree (Behlau, Canteros, Minsavage, Jones, & Graham, 2011; Gottwald et al., 2002). However, this strategy is costly and has limited effectiveness (Behlau, Canteros, Jones, & Graham, 2012; Behlau et al., 2016). Moreover, copper sprays are known to leave footprints on the environment and may help the emergence of resistant Xac strains (Canteros, 1999). Copper is not broken down in the environment and can be accumulated in plants and animals that live on copper‐contaminated soils. Therefore, there is an urgent need for environmental friendly compounds that can be used to prevent the imminent spread of Xac (and associated economic damage to citriculture), as well as to prevent the accumulation of toxic compounds in the soil.

Earlier, we and others identified alkyl gallates as broad‐spectrum antimicrobial agents that can kill Xac, *Salmonella* sp., *Staphylococcus aureus* including MRSA, *B. subtilis,* and other bacteria (Król et al., 2015; Kubo, Fujita, Ken‐ichi Nihei, & Nihei, 2004; Kubo, Fujita, & Nihei, 2002; Kubo, Fujita, Nihei, & Masuoka, 2002; Kubo, Xiao, & Fujita, 2002; Shibata et al., 2005; Silva et al., 2013). In an important manner, these compounds are not mutagenic or cytotoxic and therefore safe to use (Silva, Polaquini, Regasini, Ferreira, & Pavan, 2017). Alkyl gallates are esters of gallic acid, the main product of tannin hydrolysis. The hydrolysis of alkyl gallates produces gallic acid and the corresponding alcohols (or alkanols), which both are common components in many plants. Alkyl gallates have a head‐and‐tail structure similar to alkanols, suggesting that their antibacterial mode of action may be as surface‐active agents affecting membrane integrity (Takai, Hirano, & Shiraki, 2011). Using membrane permeability to propidium iodide as an indicator for disruption of membrane integrity, we showed that alkyl gallates indeed permeabilize the membrane and that the efficacy of this activity depends on the length of the alkyl chain (Król et al., 2015). However, elongation of Xac and *B. subtilis* exposed to alkyl gallates suggested that these compounds not only target membranes but also cell division, and we could show that this is indeed the case as these compounds inhibit ring formation of *B. subtilis* FtsZ in vivo and FtsZ polymerization and GTPase activity in vitro (Król et al., 2015; Silva et al., 2013).

To formally show that alkyl gallates target FtsZ from Xac (XacFtsZ) as well, we purified and characterized the protein and tested it in the presence of alkyl gallates. His‐tagged XacFtsZ was previously investigated in a search for single‐stranded DNA‐based compounds as anti‐canker agents (Ha, Lee, Hyun, & Yoon, 2015). To the best of our knowledge, this is the first study of fully native XacFtsZ and the GTPase activity of the protein.

## 2 MATERIALS AND METHODS

### 2.1 Cloning, protein expression, and purification

Our initial attempts at XacFtsZ expression failed (Król et al., 2015), but a close inspection of the Xac genome annotation revealed that the *ftsZ* start codon was incorrectly assigned and that the *ftsZ* gene included an additional 27 bases at the 5′ end. Thus, the Xac *ftsZ* gene was amplified from an expression vector (pCXZ‐FtsZ, a gift from H. Ferreira) lacking the upstream sequence and cloned into the pET15b vector (Novagen). The following primers were used: forward‐GAGC**CCATGG** **CACATTTCGAACTGATTGAAAAA**ATGGCTCCCAACGCGGTCATCAAGG, where the *NcoI* site is underlined and the additional 27 bases of Xac *ftsZ* are in bold; reverse‐AGTTCATATGCGACGCAGCCGACGCTCC**TCA**G, where the *NdeI* site is underlined and the stop codon is in bold. *Escherichia coli* DH5α was used as a host for DNA manipulation. The resulting plasmid, pET15b‐XacFtsZ, was sequenced to ensure that the *ftsZ* coding sequence was correct.

XacFtsZ was expressed in *E. coli* BL21 (DE3) cells containing the pET15b‐XacFtsZ vector. The cells were cultivated in LB medium containing 100 μg/ml ampicillin at 37°C until OD_600_ was 0.6–0.8 and then in LB medium containing 100 μg/ml ampicillin and 1 mM IPTG at 30°C for 4 hr. After that, the cells were harvested and stored frozen until the purification, for which they were resuspended in a TRIS50 buffer (see below). The cells were disrupted by sonication for 8 min at amplitude 7, in 8 s on/off intervals (MSE Soniprep 150) with concomitant cooling on an ice/ethanol mixture. The insoluble fraction was removed by centrifugation at 70,000*g* for 45 min at 4°C. XacFtsZ was purified by ammonium sulfate precipitation from the soluble fraction in the TRIS50 buffer. The protein precipitated at 20% of saturated ammonium sulfate, which was added gradually to the soluble fraction which was stirred gently while being kept on ice. The pellet of XacFtsZ was centrifuged at 3,200*g* for 20 min at 4°C, resuspended, and dialyzed against TRIS50 buffer in order to remove residual ammonium sulfate. This resulted in a pure (>95%) FtsZ fraction, as confirmed by SDS‐PAGE gel analysis. The protein was stored at −80°C in small aliquots, which, after defrosting, were either used immediately or after a few days of storage at 4°C. After a few days, the thawed protein was discarded, and once thawed, protein was never refrozen.

### 2.2 Analytical methods

The following buffers were used: 50 mM MES pH 6.5, 50 mM KCl (MES50); 50 mM MES pH 6.5, 300 mM KCl (MES300); 50 mM PIPES pH 6.8, 50 mM KCl (PIPES50); 50 mM PIPES pH 6.8, 300 mM KCl (PIPES300); 50 mM HEPES pH 7.0, 50 mM KCl (HEPES7.0_50); 50 mM HEPES pH 7.0, 300 mM KCl (HEPES7.0_300); 50 mM HEPES pH 7.5, 50 mM KCl (HEPES7.5_50); 50 mM HEPES pH 7.5, 300 mM KCl (HEPES7.5_300); 50 mM TRIS pH 7.9, 50 mM KCl (TRIS50); 50 mM TRIS pH 7.9, 300 mM KCl (TRIS300). Sedimentation, light scattering, and GTPase assays were performed according to literature protocols (Ingerman & Nunnari, 2006; Król & Scheffers, 2013; Margalit et al., 2004). FtsZ was polymerized at 30°C in an appropriate buffer with 5 mM MgCl_2_ and 1 mM nucleotide (or a corresponding volume of buffer) for 5 min in a water bath and for approximately 7 min until samples were spun down at 186,000*g*. GTPase activity was measured with a continuous, regenerative coupled assay that detects the decrease of NADH absorption used for the regeneration of GDP to GTP (Ingerman & Nunnari, 2006; Margalit et al., 2004). The reaction was performed at 30°C at different FtsZ concentrations in TRIS50 buffer with 5 mM MgCl_2_ and 400 *μ*M GTP, in the presence or absence of an alkyl gallate, as indicated in the results section. The assay components for GTP regeneration were 20 U/ml pyruvate kinase/lactic dehydrogenase enzymes from rabbit muscle (Sigma‐Aldrich), 1 mM NADH (Sigma‐Aldrich), and 2 mM phospho(enol)pyruvic acid monopotassium salt (Sigma‐Aldrich). The reaction was followed by the measurement of 340 nm absorbance on a BioTek Synergy^™^ Mx Microplate Reader. For the analysis of the nucleotide bound to FtsZ, the protein was precipitated with 10% TCA and the resulting supernatant was loaded on a MonoQ HR 5/5 1 ml column (GE Healthcare) in 10 mM KH_2_PO_4_/K_2_PO_4_ pH 8.0 buffer and run in a gradient formed by 50 mM mM KH_2_PO_4_/K_2_PO_4_ pH 7.4, 1M NaOH. The concentrations of the corresponding nucleotide were calculated based on reference GTP, GDP, and GMP solutions.

### 2.3 Calibration of protein concentration

Quantitative amino acid analysis of FtsZ was performed by Eurosequence (the Netherlands). Commercial BSA (Thermo Fisher) and calibrated FtsZ were used to calibrate Bradford (Thermo Fisher), bicinchoninic acid (Thermo Fisher), and DC (Bio‐Rad) colorimetric assays.

### 2.4 Electron microscopy

XacFtsZ in the polymerization buffer was incubated at 30°C, and samples were collected at different time points and applied to an electron microscopy grid as described in (Król & Scheffers, 2013). The grids were examined in a Philips CM120 electron microscope equipped with a LaB_6_ filament operating at 120 kV. Images were recorded with Gatan 4000 SP 4K slow‐scan CCD camera.

### 2.5 Membrane permeability assay

Membrane permeability was essentially assayed as described (Król et al., 2015) with the following modifications for Xac: 100 μl of aliquots of exponentially growing Xac (OD600 ≈ 0.5, 30°C, NYGB medium: peptone 5 g/L; yeast extract 3 g/L; glycerol 20 g/L (Lorenzoni, Dantas, Bergsma, Ferreira, & Scheffers, 2017)) was incubated for 45 min with either alkyl gallates at MIC_50_ (31.2 μg/ml; (Silva et al., 2013)) or Nisin (10 μg/ml) and EDTA (30 mM) as a control for permeabilization, and nothing for a negative control. After 45 min, 0.3 μl of dye mixture (propidium iodide (5.0 mM) and SYTO 9 (835 μM), LIVE/DEAD^®^ BacLight^™^ Bacterial Viability Kit, ThermoFisher Scientific, used as described by manufacturer) was added to the cells to stain the DNA—incubation was continued for 15 min and immediately imaged on agarose pads on bright‐field, and fluorescence filters FITC (50 ms) and TRITC (100 ms). Images were analyzed on Fiji (https://fiji.sc/); the function “Find Maxima” was used to automatically detect green and red fluorescent cells with thresholds of 11,000 and 3,000, respectively.

## 3 RESULTS

### 3.1 Native Xac FtsZ production and purification

In order to produce and purify FtsZ from *Xanthomonas* in a soluble form, the Xac *ftsZ* gene was cloned into the pET15b vector, which should allow production of the native protein. XacFtsZ proved to be expressed as soluble protein in *E. coli* BL21 cells and ammonium sulfate precipitation was sufficient to obtain the protein that did not need any further purification steps, as established by SDS‐PAGE analysis (not shown). This provides a very fast and cheap purification of XacFtsZ that can be produced in high yield (about 100 mg of pure protein from half a liter of LB medium). To our knowledge, this is the first time when XacFtsZ was obtained without any purification tag.

**Figure 1:**
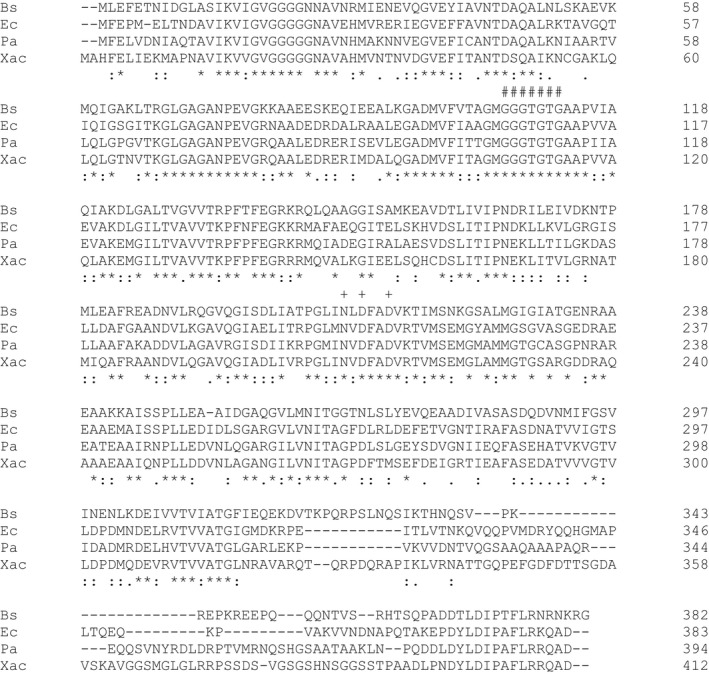
Amino acid sequence alignment of XacFtsZ with FtsZs from *Bacillus subtilis* (Bs), *Escherichia coli* (Ec), and *Pseudomonas aeruginosa* (Pa). Certain conserved residues are marked: #, tubulin signature motif; +, hydrolytic residues. Prepared using ClustalW

FtsZ proteins have a quite unique amino acid composition, with a very low number of aromatic amino acids—they contain no tryptophans and only a few tyrosines (XacFtsZ only one, Figure 1). Therefore, the absorbance at 280 nm is not an accurate method to determine the concentration of the protein. Various colorimetric methods produce a certain error that is related to the colorimetric reaction detecting, for example, aromatic amino acids or cysteines (of which XacFtsZ only contains two). In a traditional manner, the concentration of FtsZ from different species was assessed by calibrating a number of colorimetric assays against the standard protein, which is normally BSA (Lu & Erickson, 1998; Lu, Stricker, & Erickson, 1998). Here, we performed a quantitative amino acid analysis of FtsZ that was further used to calibrate three colorimetric assays against commercial BSA. This gave the following correction factors: 0.60 for the Bradford assay, 0.69 for the BCA assay, and 0.76 for the DC assay. We decided to use the Bradford assay as our standard method to determine FtsZ concentration, due to its reproducibility and simplicity.

### 3.2 XacFtsZ is a true GTPase

XacFtsZ, together with its eukaryotic homolog tubulin, belongs to a family of protofilament‐forming GTPases (Löwe & Amos, 1999; Nogales, Downing, Amos, & Löwe, 1998). A Blast search shows that FtsZ from *Pseudomonas aeruginosa*,* Pseudomonas putida,* and *Azotobacter vinelandii* are the closest XacFtsZ homologs. ClustalW alignment (Figure 1) shows the following sequence identities and similarities, respectively, with XacFtsZ: *P. aeruginosa* (Pa) 61.0% and 75.1%, *P. putida* 60.6% and 76.0%, *A. vinelandii* 60.1% and 75.2%, *E. coli* (Ec) 57.0% and 70.2%, and *B. subtillis* (Bs) 44.4% and 63.3%. In addition to the tubulin signature motif (GGGTGS/TG) (de Boer, Crossley, & Rothfield, 1992), XacFtsZ contains all the three highly conserved amino acids responsible for the GTPase activity: N210, D212, and D215 (corresponding to N207, D209, and D212 in *E. coli*).

**Figure 2:**
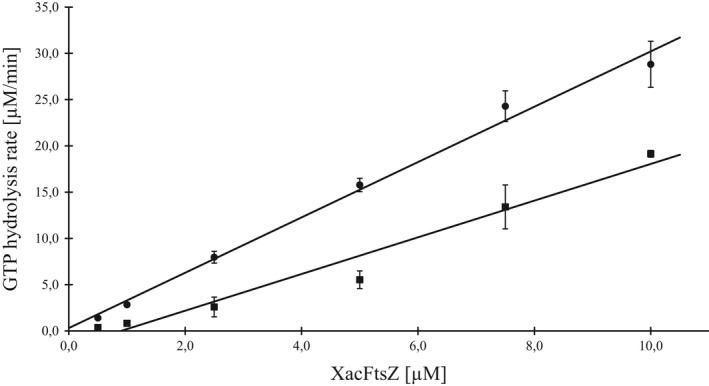
The critical concentration of XacFtsZ in the absence (dots) and in the presence of 50 μg/ml heptyl gallate (squares) assayed by the GTPase activity at 30°C. The data were fitted with the linear equation resulting in: *y* = 2.9914*x* + 0.3013 (*R* ^2^ = 0.9922) for free XacFtsZ and *y* = 1.9832*x* – 1.7847 (*R* ^2^ = 0.9653) for XacFtsZ in the presence of heptyl gallate. The data show the mean of at least four repeats. Error bars represent standard deviation

In order to confirm that XacFtsZ is a true GTPase, the hydrolysis activity of the purified protein was measured with a continuous, regenerative coupled assay that detects the decrease of NADH absorption used for the regeneration of GDP to GTP (Ingerman & Nunnari, 2006; Margalit et al., 2004). The reaction was performed at 30°C at different FtsZ concentrations (Figure 2). The observed rate of 3 GTP/min per molecule of FtsZ is typical for FtsZ proteins (Buske & Levin, 2012; Milam & Erickson, 2013; Mukherjee & Lutkenhaus, 1999; Sundararajan et al., 2015), and the increase in activity was linear in the chosen concentration range. It is interesting that there was no decrease in the activity at low FtsZ concentration and the extrapolated *x*‐intercept is very close to 0. This suggests a very low apparent critical concentration compared to FtsZ proteins from different species, which is normally in the range of 1–3 *μ*M (Chen, Milam, & Erickson, 2012; Hernández‐Rocamora, Alfonso, Margolin, Zorrilla, & Rivas, 2015; Mukherjee & Lutkenhaus, 1999; Oliva et al., 2003; White et al., 2000; Yang et al., 2016). The type of buffer (TRIS50 or HEPES7.5_50) and magnesium concentration (5–10 mM) did not influence XacFtsZ activity significantly (data not shown).

### 3.3 XacFtsZ polymerizes independently of external GTP

**Figure 3:**
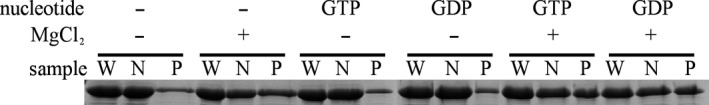
Sedimentation assay of XacFtsZ. FtsZ was polymerized for approx. 12 min (5 min in water bath + ~7 min until samples started to spin down at 186,000*g*). 5.2 μM FtsZ was polymerized at 30°C in TRIS50 buffer with 5 mM MgCl_2_ and 1 mM nucleotide (or a corresponding volume of buffer), where indicated. W—whole sample input, N—supernatant fraction and P—pellet fraction

In vivo, FtsZ exists in an equilibrium between monomers and polymers. This equilibrium is dynamic and controlled by many protein and nonprotein factors influencing cell division, but also the general metabolism of a cell (Adams & Errington, 2009; Adams, Wu, & Errington, 2014; Addinall, Cao, & Lutkenhaus, 1997; Erickson, Anderson, & Osawa, 2010; Lan, Wolgemuth, & Sun, 2007; Stricker, Maddox, Salmon, & Erickson, 2002; Sun & Margolin, 1998). In vitro however, FtsZ is able to polymerize in certain simplified conditions that usually require GTP that is bound between monomers of the polymer in the presence of magnesium ions. The amount of polymers formed is traditionally quantified in a sedimentation assay (Bramhill & Thompson, 1994). In a surprising manner, XacFtsZ showed a significant pellet in the absence of any of the two nucleotides, GTP and GDP, as long as magnesium was present (Figure 3). This was not the case if this ion was absent, even if GTP or GDP was added. The most significant pellets were formed in the presence of magnesium and any of the two nucleotides, but there was no striking difference in the amount of pellet formed in the presence of GTP or GDP.

**Figure 4:**
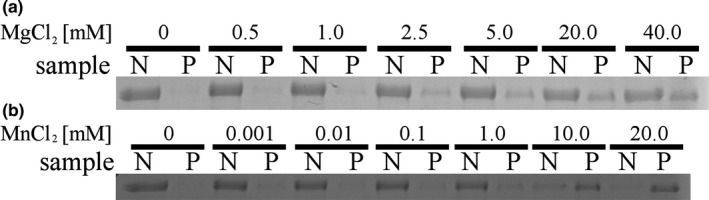
Influence of magnesium (a) and manganese (b) on FtsZ sedimentation. The procedure was performed as in Figure 3. N—supernatant fraction and P—pellet fraction, input samples were not loaded on SDS‐PAGE

This atypical behavior prompted us to hypothesize that XacFtsZ copurifies with a GTP nucleotide bound to the protein, which would allow polymerization to be triggered by the addition of magnesium ions, as shown before for certain mutants of *E. coli* FtsZ (Scheffers, de Wit, den Blaauwen, & Driessen, 2001). Therefore, nucleotides that copurified with XacFtsZ were extracted from the protein with 10% TCA and analyzed using FPLC with the use of GTP, GDP, and GMP as standard references. Purified XacFtsZ had 0.9 mol of bound GDP (but no GTP or GMP) per 1 mol of protein, which makes it similar to FtsZ from *E. coli* (RayChaudhuri & Park, 1992; Scheffers et al., 2001; Zorrilla, Minton, Vicente, & Andreu, 2000). Furthermore, the amount of added magnesium influenced the amount of XacFtsZ pelleted in the sedimentation assay (Figure 4). In the absence of any external nucleotide, the pellet started to be visible between 0.5 and 1.0 mM of MgCl_2_ and continued to increase above 20 mM MgCl_2_. To see whether this effect was specific for Mg^2+^ or also occurred with other divalent cations, we used Mn^2+^ which, as we previously showed, stabilizes BsFtsZ polymers (Król, de Sousa Borges, Kopacz, & Scheffers, 2017). The effect was similar when XacFtsZ was allowed to polymerize in the presence of MnCl_2_, but here a lower concentration was needed for the pellet to be visible and the protein was almost fully sedimented already at 10 mM MnCl_2_.

**Figure 5:**
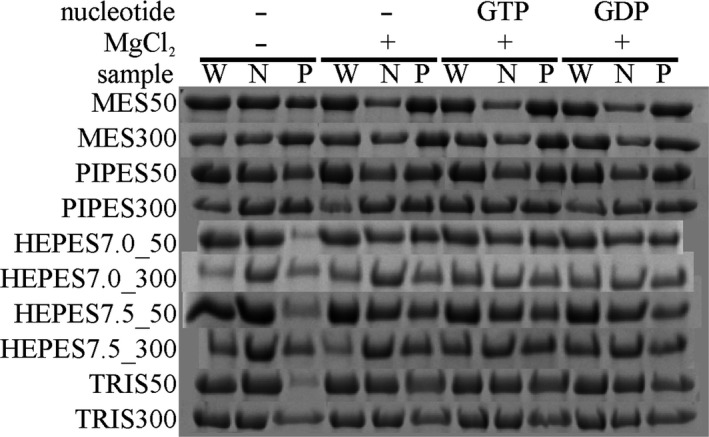
Sedimentation properties of Xac FtsZ in different buffers. The procedure was performed as in Figure 3; buffer composition is listed in materials and methods. W—whole sample input, N—supernatant fraction and P—pellet fraction. This image is (evidently) composed of pictures of different SDS‐PAGE gels and thus a composite image. Every image for a buffer condition is composed of a continuous gel segment

In addition, the sedimentation properties of XacFtsZ were analyzed in different buffers (see Materials and Methods and Figure 5). In all buffers of pH lower than 7.0, significant amounts of pellet were observed in the buffer only. Considerable pellets were also formed in the buffers of higher pH when salt concentrations were high. It was concluded that the protein is not stable in these conditions. On the other hand, the behavior of XacFtsZ was fairly similar in HEPES and TRIS buffers at 50 mM KCl, independently of the pH.

### 3.4 XacFtsZ forms very short polymers

**Figure 6:**
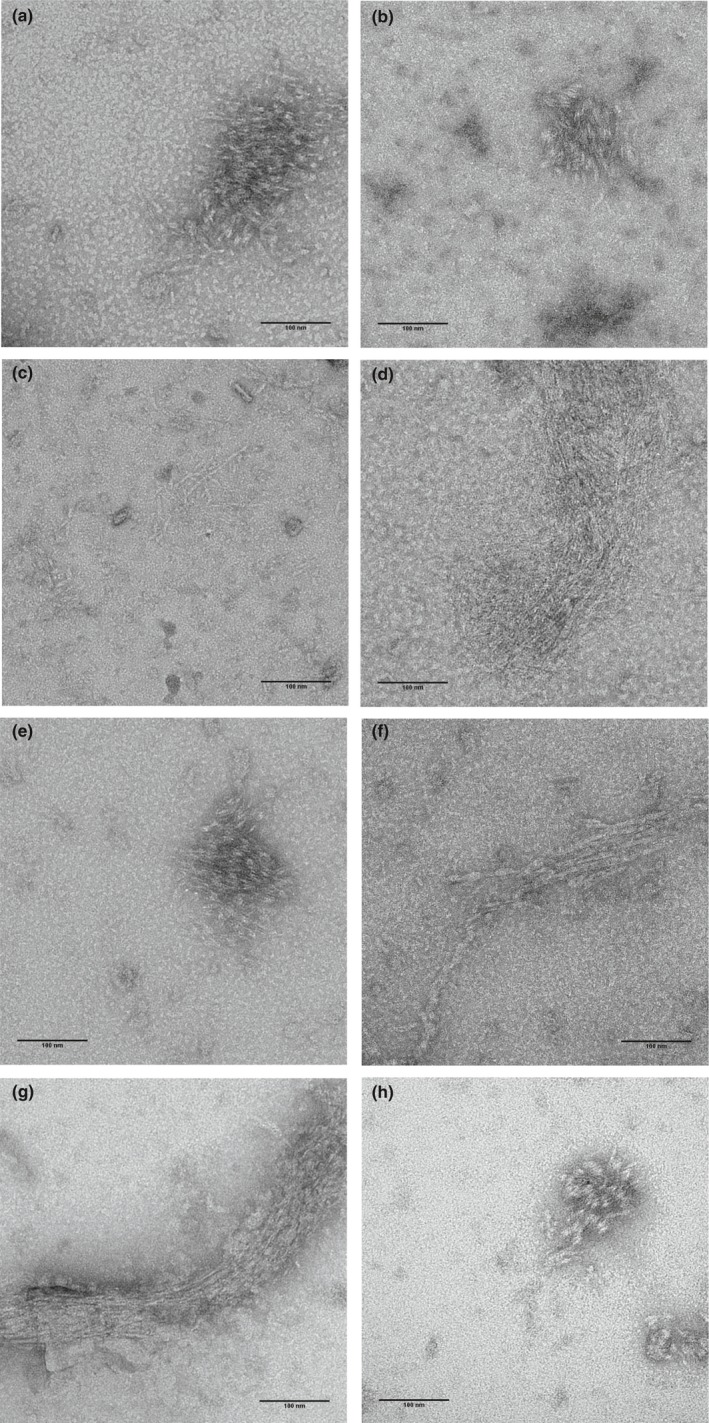
XacFtsZ forms short polymers that tend to bundle on the EM grid. 2.6 μM or 5 μM FtsZ in TRIS50 with 10 mM MgCl_2_ and 1 mM GTP was polymerized at 30°C for 0 min (a) 2 min, (b) 10 min, (c) 20 min, (d) 40 min (e), 60 min (f), 120 min (g). A sample without added GTP and incubated at 30°C for 2 min is shown in (H). While the polymers were found at different time points, they were the most abundant after 2 min of polymerization and almost not present at time 0 min

The morphology and dynamics of the polymers formed by XacFtsZ were observed with transmission electron microscopy (Figure 6) and static light scattering. The polymers were rather short, but straight when compared to FtsZ from other species (Buske & Levin, 2012; Chen et al., 2012; Król & Scheffers, 2013; Pacheco‐Gómez, Roper, Dafforn, & Rodger, 2011; Scheffers, 2008). Immediately upon addition of MgCl_2_ and GTP (Figure 6a), only a small amount of very short polymers was observed that did not increase significantly in length in time. The overall amount of polymers was much bigger already after short incubation of 2 min (Figure 6b), and they tended to form large clusters. At longer incubation times (Figure 6c–g), the morphology was generally the same, but the polymers were gradually less abundant and a few circular forms of different diameters were found. However, we do not know whether they are of any physiological importance. It is important that incubation with MgCl_2_ alone also resulted in the formation of short polymer clusters (Figure 6h) although these were less abundant and shorter than what was observed with GTP. This corresponds with our observation that addition of MgCl_2_ alone is sufficient to sediment XacFtsZ. It is not possible to make statements about whether there is a qualitative difference in the nature of the polymers formed with only MgCl_2_ (with FtsZ predominantly containing GDP) or after addition of GTP. Therefore, we do not draw conclusions about which nucleotide is present in XacFtsZ filaments.

FtsZ polymerization properties were tested with a static light scattering method in many different conditions, where buffer, GTP, magnesium, and protein concentrations varied. However, we were never able to observe the characteristic FtsZ polymerization and depolymerization peak, even when divalent cations (Ca^2+^, Mn^2+^) or the poly‐cation DEAE‐dextran were added to promote bundling. This could be due to the very short length of polymers formed by XacFtsZ that were observed in the electron microscopy experiments.

### 3.5 Alkyl gallates are mild inhibitors of XacFtsZ

Alkyl gallates were recently shown to be able to act as broad‐spectrum antimicrobial agents that can kill Xac (Silva et al., 2013). This activity can be attributed to the surfactant properties of alkyl gallates that disrupt the bacterial membrane; however, several alkyl gallates are also able to interact directly with FtsZ from *B. subtillis* (BsFtsZ) and cluster the protein resulting in inhibition of GTPase activity and bundling of FtsZ polymers (Król et al., 2015). Therefore, we wanted to examine whether alkyl gallates act similarly on XacFtsZ.

**Figure 7:**
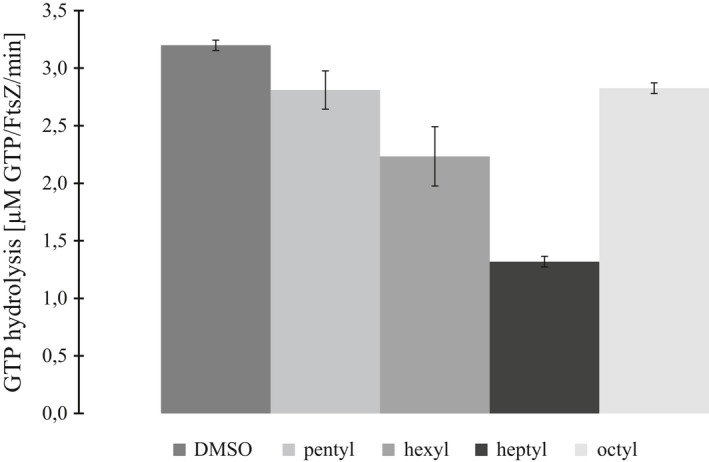
XacFtsZ GTPase activity in the presence of alkyl gallates, measured at 30°C in TRIS50 in the presence of 5 mM MgCl_2_, 400 μM GTP (constant), and 50 μg/ml alkyl gallate. The reaction was started with the addition of 5 μM protein. The reaction at 1% DMSO (vehicle solvent) was used as a control. The data are a mean of four repeats, and the bars represent standard deviation

In a previous study, pentyl, hexyl, heptyl, and octyl gallates were selected for testing with BsFtsZ, based on their ability to elongate Xac cells and disrupt their cell division machinery (Król et al., 2015; Silva et al., 2013). In the presence of 50 μg/ml of either alkyl gallate, only residual GTPase activity of BsFtsZ was detected, resulting in a nearly sixfold decrease compared to the control performed in the absence of the alkyl gallate. This was not the case with XacFtsZ, where a reduction in activity was found, but less significant and differing between the alkyl gallates used. The presence of hexyl and heptyl gallate caused approximately 30% and 60% decrease in XacFtsZ activity, while pentyl and octyl gallate showed only approximately 15% of reduction (Figure 7).

Because heptyl gallate showed the strongest inhibition of XacFtsZ GTPase activity, the activity was also measured at variable concentrations of XacFtsZ, while keeping the concentration of heptyl gallate constant (Figure 2). The apparent critical concentration of XacFtsZ shifted to approximately 1 μM, indicating that heptyl gallate binds to the monomers of XacFtsZ, excluding them from the pool for polymerization. The high concentration of heptyl gallate (50 μM), compared to the one of XacFtsZ (0.5–10 μM), suggests that heptyl gallate has a rather low affinity toward the protein. Furthermore, the difference in slope of the two fits indicates that XacFtsZ is inhibited in the polymerized form as well.

**Figure 8:**
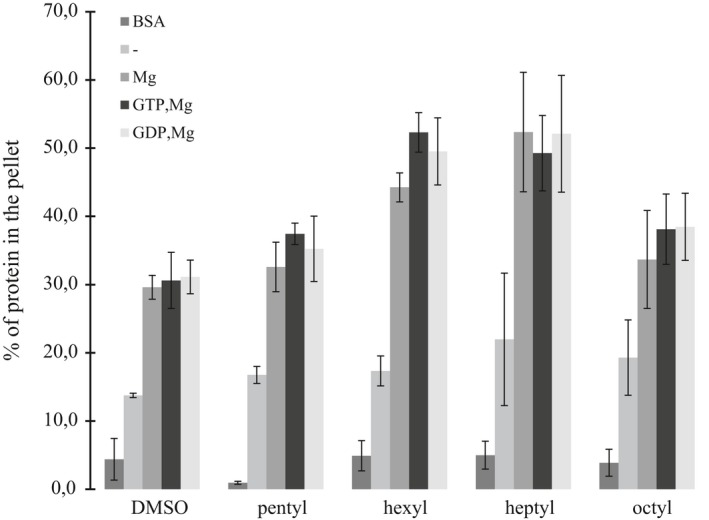
The size of the sedimented pellet of XacFtsZ in the presence of alkyl gallates. 10 μM XacFtsZ or BSA (control) was sedimented as in Figure 3 in the presence of 100 μg/ml of alkyl gallate or 1% DMSO (vehicle solvent). The percentage of pelleted protein (BSA or FtsZ) was calculated with ImageJ from SDS‐PAGE analysis. The data are a mean of three repeats, and the bars represent standard deviation

In order to see how alkyl gallates influence assembly of XacFtsZ, we performed the sedimentation assay in the presence of 100 μg/ml of each alkyl gallate (Figure 8). Due to the ability of the protein to sediment in the sole presence of magnesium ions, we allowed XacFtsZ to polymerize in the absence and in the presence of the cation, as well as in the presence of the cation and one of the nucleotides, GTP or GDP. The results corroborated with the XacFtsZ GTPase activity: Pentyl and octyl gallates showed minimal influence on the sedimentation of the protein, while in the presence of hexyl or heptyl gallate XacFtsZ sedimented the most. In addition, the pellet formation was increased by the alkyl gallates in the presence of magnesium and the presence of nucleotide had only a minor effect. The compounds may induce XacFtsZ clustering or aggregation because the pellet fraction was also higher in the absence of magnesium. However, this interaction seems to be XacFtsZ specific, because the compounds did not influence the pellet formation in the case of BSA.

### 3.6 Alkyl gallates permeabilize Xac membranes

As the inhibitory effect of the alkyl gallates on XacFtsZ was relatively mild, we analyzed whether, as in the Gram‐positive *B. subtilis*, alkyl gallates can disrupt Xac membranes. A permeabilization assay clearly showed that at MIC_50_ pentyl gallate permeabilized 42% of Xac cells, and the other alkyl gallates permeabilized 94% or more Xac cells (Table 1). This shows that the antibacterial activity of the alkyl gallates against Xac is primarily caused by the effect of the alkyl gallates on the membranes. We recently reported a similar membrane permeabilization for mono‐acetylated versions of these alkyl gallates (Savietto et al., 2018).

**Table 1:** Permeabilization of Xac cells by alkyl gallates

Condition (n/n)	Permeabilized cells (mean ± *SD*)
Control (10,536/10,010)	0.8 ± 0.2%
Pentyl gallate (308/268)	42.2 ± 0.3%
Hexyl gallate (457/858)	94.5 ± 4.2%
Heptyl gallate (1,931/5,701)	99.9 ± 0.1%
Octyl gallate (1,523/3,508)	99.8 ± 0.1%
Nisin EDTA (452/5,437)	94.0 ± 5.9%

Results from two independent replicates, n represents the number of cells counted in each experiment.

## 4 CONCLUSION

Here, we have purified and studied native FtsZ from Xac. Although XacFtsZ purifies and displays GTPase activity comparable to FtsZs from model organisms such as *E. coli* and *B. subtilis*, its polymerization properties are distinctly different. Addition of divalent metal ions proved sufficient to sediment XacFtsZ, which forms short polymers observable by EM. This was not due to retention of GTP in the active site of purified XacFtsZ as the predominant nucleotide associated with the purified protein was GDP. This suggests that XacFtsZ polymers are relatively stable, compared to other FtsZs, in the GDP‐form. There is one study in the literature on XacFtsZ biochemistry, in which polymerization is followed by static light scattering (Ha et al., 2015). We have not been able to reproduce this result but note that the protein purified by Ha et al. contains a C‐terminal His‐tag (Ha et al., 2015). It is well documented that the charge at the C‐terminal tail of FtsZ is important to mediate electrostatic interactions that drive bundling of FtsZ polymers (Buske & Levin, 2012). A positive charge at the C‐terminus is a strong stimulator of bundling (Buske & Levin, 2012), and we presume that the presence of the hexa‐histidine tag in combination with the slightly acidic pH of 6.5, which was used by Ha et al., explains why these authors observed polymerization by static light scattering, which we could not reproduce with native XacFtsZ. Using GTPase activity as a robustly reproducible and quantitative indicator of FtsZ activity, we were able to show that XacFtsZ is inhibited by alkyl gallates although not to a similar extent as *B. subtilis* FtsZ. Although treatment of Xac with alkyl gallates caused cell elongation (Silva et al., 2013), filamentation of cells to more than double the length of the cell, as expected for a true cell division inhibitor, was not found for Xac, and only at reduced concentrations of alkyl gallates in *B. subtilis* (Król et al., 2015). Combined with our observation that Xac membranes are permeabilized by the alkyl gallates, we conclude that the killing of Xac by alkyl gallates, especially at concentrations around MIC_50_ or higher, is primarily mediated through membrane permeabilization, not by its inhibition of XacFtsZ.

## CONFLICT OF INTEREST

The authors declare no conflict of interest.

## Data Availability

All data are included in the main manuscript. Raw data and materials are available on request.
